# Supplementary material for: Optimizing Stability in Dynamic Small-Molecule Binding Proteins
Source: J Am Chem Soc. 2025 Dec 29;148(1):1911–8. doi: 10.1021/jacs.5c19571 (PMC12814167; doi:10.1021/jacs.5c19571)
Supplement: Supplementary file 1 [file ja5c19571_si_001.pdf]

Supporting Information

Optimizing stability in dynamic small-molecule binding proteins

Marc Scherer [1,2], Mark Kriegel [1], Birte Höcker [1,*], Sarel J. Fleishman [2,*]

[1] University of Bayreuth, Department of Biochemistry, Bayreuth, Germany

[2] Weizmann Institute of Science, Department of Biomolecular Sciences, Rehovot, Israel

Supporting Tables

Supporting Table 1 - Original organism and PDB identifiers of the PBPs

Supporting Table 2 - Amino acid sequences of PBP wild types and designs

Supporting Table 3 - Primers used for cloning

Supporting Table 4 - Thermal melt data of PBP wild types and designs

Supporting Table 5 - Protein and ligand concentrations for ITC measurements

Supporting Table 6 - ITC data of PBP wild types and designs

Supporting Figures

Supporting Figure 1 - FilterScan $\Delta\Delta G$ values of PROSS mutations categorized by layer

Supporting Figure 2 - PBP interface residues fixed in PROSS design

Supporting Figure 3-6 - Amino acid sequence alignment of PBP designs

Supporting Figure 7 - CD spectra of PBP wild types and designs

Supporting Figure 8 - CD thermal melts of PBP wild types and designs without ligand

Supporting Figure 9 - CD thermal melts of PBP wild types and designs with ligand

Supporting Figure 10-13 - ITC data of PBP wild types and designs

Protein target	Original organism	PDB ID (open)	PDB ID (closed)
PotF	Escherichia coli (strain K12)	6YED [1]	6YE0 [1]
TphC	Comamonas sp.	7NDR [2]	7NDS [2]
MBP	Escherichia coli (strain K12)	1OMP [3]	3MBP [4]
LAO	Salmonella typhimurium (strain LT2/SGSC1412/ATCC 700720)	2LAO [5]	1LAF [6]

Supporting Table 1. Original organism and PDB[7] identifiers of the PBP crystal structures used.

Protein target	Design ID	Amino acid sequences
PotF	PotF.0	MAEQKTLHIYNWSDYIAPDTVANFEKETGIKVVYDVFDSNEVLEGKLMAGSTGFDLV<br>VPSASFLERQLTAGVFQPLDKSKLPEWKNLDPELLKLVAKHDPDNKFAMPYMWATTG<br>IGYNVDKVKAVLGENAPVDSWDLILKPENLEKLKSCGVSFLDAPEEVFATVLNYLGK<br>DPNSTKADDYTGPATDLLLKLRPNIRYFHSSQYINDLANGDICVAIGWAGDVWQASN<br>RAKEAKNGVNVSFSIPKEGAMAFFDVFAMPADAKNKDEAYQFLNYLLRPDVVAHISD<br>HVFYANANKAATPLVSAEVRENPGIYPPADVRAKLFTLKVQDPKIDRVRTRAWTKVK<br>SGKLEHHHHHH
	PotF.1	MAEQKTLHIYNWSDYIAPDTIANFEKETGIKVVYDVFDSNEVLEGKLMAGSTGFDLV<br>VPSASFLERQLTAGVFQPLDKSKLPNWKNLDPELLKLVAQHDPDNKYAIPYMWATTG<br>IGYNVDKVKAVLGEDAPVDSWDLILKPENLEKLKSCGVAFLDAPEEIFATVLNYLGK<br>DPNSTNPDDYTGPATDLLLKLRPNIRYFHSSQYINDLANGDICVAIGWAGDVWQAAN<br>RAKEAKNGVNIAYTIPKEGAMAFFDVFAMPADAKNKDEAYQFLNYLLRPDVMAHISD<br>HVYYANANKAALPLVNPEVRDNPGIYPPPDVRAKLFTLKVQDPKIDRVRTRAWTKVK<br>SGKLEHHHHHH
	PotF.2	MAEQKTLHIYNWSDYIAPDTVANFEKETGIKVVYDVFDSNEVLEGKLMAGSTGFDLV<br>VPSASFLERQLTAGVFQPLDKSKLPNWKNLDPELLKLVAKHDPDNKYAMPYMWATTG<br>IGYNVDKVKAVLGEDAPVDSWDLILKPENLEKLKSCGVAFLDAPEEIFATVLNYLGK<br>DPNSTNADDYTGPATDLLLKLRPNIRYFHSSQYINDLANGDICVAIGWAGDVWQAAN<br>RAKEAKNGVNVAYSIPKEGAMAFFDVFAMPADAKNKDEAYQFLNYLLRPDVVAHISN<br>HVFYANANKAATSLVSAEVRDNPGIYPPPDVRAKLFTLKVQDPKIDRVRTRAWTKVK<br>SGKLEHHHHHH
	PotF.3	MAEQKTLHIYNWSDYIAPDTVANFEKETGIKVVYDVFDSNEVLEGKLMAGSTGFDLV<br>VPSASFLERQLSAGVFQPLDKSKLPNWKNLDPELLKLVAKHDPDNKYAMPYMWATTG<br>IGYNVDKVKAVLGEDAPVDSWDLILKPENLEKLKSCGVAFLDAPEEIFATVLNYLGK<br>DPNSTNADDYTGPATDLLLKLRPNIRYFHSSQYINDLANGDICVAIGWAGDVWQAAN<br>RAKEAKNGVNVAYSIPKEGAMAFFDVFAMPADAKNKDEAYQFLNYLLRPDVVAHISD<br>HVFYANANKAATPLVSAEVRDNPGIYPPPDVRAKLFTLKVQDPKIDRVRTRAWTKVK<br>SGKLEHHHHHH
	PotF.4	MAEQKTLHIYNWSDYIAPDTVANFEKETGIKVVYDVFDSNEVLEGKLMAGSTGFDLV<br>VPSASFLERQLTAGVFQPLDKSKLPNWKNLDPELLKLVAKHDPDNKYAMPYMWATTG<br>IGYNVDKVKAVLGEDAPVDSWDLILKPENLEKLKSCGVAFLDAPEEIFATVLNYLGK<br>DPNSTNADDYTGPATDLLLKLRPNIRYFHSSQYINDLANGDICVAIGWAGDVWQAAN<br>RAKEAKNGVNVAFSIPKEGAMAFFDVFAMPADAKNKDEAYQFLNYLLRPDVVAHISD<br>HVFYANANKAATALVSAEVRENPGIYPPADVRAKLFTLKVQDPKIDRVRTRAWTKVK<br>SGKLEHHHHHH
TphC	TphC.0	MGSSHHHHHHGSGENLYFQSNQPLKIVVPFSAGGTADVLPRLVAEKIRADYAGGVII<br>ENKPGAGGNIGADLVFRAPPDGMTVLASPPGPIAINHNLYQKLSFDPTRWVPVTILA<br>TVPNVLVINPKLPVKSLGEFIAYAKANPKKVTVATQGDGSTSHLTAAMFMQLTGTEL<br>TVIPYKGTAPALIDLIGGNVDVFFDNISSSATYHQAGKVRILAVADEQRSQILPQVP<br>TFAEQQWPAMQAVTFFSVVAPPGTSAEIAQKLQKQMALALSSNDIRKHFQEQGAVPC<br>GWDPSKTAQFIRQETEKWKKVLKAANVKL
	TphC.1	MGSSHHHHHHGSGENLYFQSNQPLKIVVPFSPGGTADVLPRLVAEKIRADYGGGVII<br>ENKPGAGGNIGADLVFRAPPDGYTVLISPPGPIAINHNLYKKLSFDPTRWVPVTILA<br>TVPNVLVINPKLPVKSVKEFIAYAKANPGKLTVATQGNGSTSHLTAELFMQMTGTDM<br>THIPYKGTAPALIDLIGGNVDVFFDNISSSATYHQAGKVRILAVADEQRSPILPDVP

		TFAEQGFPAFQVVTFFSVVAPPGTSAEIVQKLQKQIAKALSSPDIRKHFQEQGAVPV<br>GWDPSQTAQFIRQETEKWKKVIKAANVKL
	TphC.2	MGSSHHHHHHGSGENLYFQSNQPLKIVVPFSPGGTADVLPRLVAEKIRKDYAGGVII<br>ENKPGAGGNIGADLVFRAPPDGMTVLASPPGPIAINHNLYKKLSFDPTRWVPVTILA<br>TVPNVLVINPKLPVKSVKEFIAYAKANPGKVTVATQGNGSTSHLTAAMFMQLTGTDM<br>THIPYKGTAPALIDLIGGNVDVFFDNISSSAQYHQAGKVRILAVADEQRSPILPDVP<br>TFAEQGFPAMQAVTFFSVVAPPGTSAEIAQKLQKQMAKALSSPDIRKHFQEQGAVPV<br>GWDPEQTAQFIRQETEKWKKVLKAANVKL
	TphC.3	MGSSHHHHHHGSGENLYFQSNQPLKIVVPFSPGGTADVLPRLVAEKIRADYAGGVII<br>ENKPGAGGNIGADLVFRAPPDGMTVLASPPGPIAINHNLYKKLSFDPTRWVPVTILA<br>TVPNVLVINPKLPVKSVKEFIAYAKANPGKVTVATQGNGSTSHLTAAMFMQLTGTDM<br>THIPYKGTAPALIDLIGGNVDVFFDNISSSATYHQAGKVRILAVADEQRSPILPDVP<br>TFAEQGFPAMQAVTFFSVVAPPGTSAEIAQKLQKQMAKALSSPDIRKHFQEQGAVPV<br>GWDPSQTAQFIRQETEKWKKVLKAANVKL
	TphC.4	MGSSHHHHHHGSGENLYFQSNQPLKIVVPFSAGGTADVLPRLVAEKIRADYAGGVII<br>ENKPGAGGNIGADLVFRAPPDGYTVLASPPGPIAINHNLYQKLSFDPTRWVPVTILA<br>TVPNVLVINPKLPVKSLKEFIAYAKANPGKVTVATQGDGSTSHLTAAMFMQLTGTEL<br>TVIPYKGTAPALIDLIGGNVDVFFDNISSSATYHQAGKVRILAVADEQRSQILPQVP<br>TFAEQGFPAMQAVTFFSVVAPPGTSAEIAQKLQKQMAKALSSPDIRKHFQEQGAVPC<br>GWDPEQTAQFIRQETEKWKKVLKAANVKL
MBP	MBP.0	MKIEEGKLVIWINGDKGYNGLAEVGKKFEKDTGIKVTVEHPDKLEEKFPQVAATGDG<br>PDIIFWAHDRFGGYAQSGLLAEITPDKAFQDKLYPFTWDAVRYNGKLIAYPIAVEAL<br>SLIYNKDLLPNPPKTWEEIPALDKELKAKGKSALMFNLQEPYFTWPLIAADGGYAFK<br>YENGKYDIKDVGVDNAGAKAGLTFLVDLIKNKHMNADTDYSIAEAAFNKGETAMTIN<br>GPWAWSNIDTSKVNYGVTVLPTFKGQPSKPFVGVLSAGINAASPNKELAKEFLENYL<br>LTDEGLEAVNKDKPLGAVALKSYEEELAKDPRIAATMENAQKGEIMPNIPQMSAFWY<br>AVRTAVINAASGRQTVDEALKDAQTRITKLEHHHHHH
	MBP.1	MKIEEGKLVIWINGDKGYNGLAEVGKKFEKDTGIKVTVEHPDKLEEKFAQVAATGDG<br>PDIIFWAHDRFGGWAQSGLLAEIHPDKEFQDKLFPFTWDAVRYNGKLIAYPIAVEAL<br>SLIYNKDLLPNPPKTWEELPELDKKLRAKGKSAIMFNLQEPYFTWPLIAADGGYAFK<br>YENGKYDIKDVGVNNEGAKAGLQFLVDLIKNKHMNADIDYSIAEAAFNKGETAMTIN<br>GPWAWSNIDKAKINYGVTVLPTFKGKPSKPFVGVLSAGINAASPNKELAKEFLENYL<br>LTDEGLDLVNKDKPLGAVALKSYQEELAKDPRIAATMENAQNGEIMPNIPQMSAFWY<br>AMRTAVINALSGRQSVDEALKDAQTRITKLEHHHHHH
	MBP.2	MKIEEGKLVIWINGDKGYNGLAEVGKKFEKDTGIKVTVEHPDKLEEKFTQVAATGDG<br>PDIIFWAHDRFGGWAQSGLLAEIHPDKAFQDKFYPFTWDAVRYNGKLIAYPVAVEAL<br>SLIYNKDLLPNPPKTWEELPELDKELRKKGKSALMFNLQEPYFTWPLIAADGGYAFK<br>YENGKYDIKDVGVNNEGAKAGLQFLVDLIKNKHMNADIDYSIAEAAFNKGETAMTIN<br>GPWAWSNIDKAKINYGVTVLPTFKGKPSKPFVGVLTAGINAASPNKELAKEFLENYL<br>LTDEGLDMVNKDKPLGAVALKSYQEELAKDPRIAATMENAQNGEIMPNIPQMSAFWY<br>AMRTAVINALSGRQTVDEALKDAQKRITKLEHHHHHH
	MBP.3	MKIEEGKLVIWINGDKGYNGLAEVGKKFEKDTGIKVTVEHPDKLEEKFAQVAATGDG<br>PDIIFWAHDRFGGWAQSGLLAEIHPDKAFQDKLYPFTWDAVRYNGKLIAYPIAVEAL<br>SLIYNKDLLPNPPKTWEELPELDKELRAKGKSALMFNLQEPYFTWPLIAADGGYAFK<br>YENGKYDIKDVGVNNEGAKAGLQFLVDLIKNKHMNADIDYSIAEAAFNKGETAMTIN<br>GPWAWSNIDKAKINYGVTVLPTFKGKPSKPFVGVLSAGINAASPNKELAKEFLENYL<br>LTDEGLDAVNKDKPLGAVALKSYQEELAKDPRIAATMENAQNGEIMPNIPQMSAFWY<br>AMRTAVINALSGRQTVDEALKDAQTRITKLEHHHHHH
		MKIEEGKLVIWINGDKGYNGLAEVGKKFEKDTGIKVTVEHPDKLEEKFAQVAATGDG<br>PDIIFWAHDRFGGYAQSGLLAEITPDKAFQDKFYPFTWDAVRYNGKLIAYPIAVEAL

	MBP.4	SLIYNKDLLPNPPKTWEELPALDKELRAKGKSALMFNLQEPYFTWPLIAADGGYAFK<br>YENGKYDIKDVGVNNEGAKAGLQFLVDLIKNKHMNADTDYSIAEAAFNKGETAMTIN<br>GPWAWSNIDKAKINYGVTVLPTFKGKPSKPFVGVLSAGINAASPNKELAKEFLENYL<br>LTDEGLDAVNKDKPLGAVALKSYQEELAKDPRIAATMENAQKGEIMPNIPQMSAFWY<br>AMRTAVINALSGRQTVDEALKDAQTRITKLEHHHHHH
LAO	LAO.0	MALPQTVRIGTDTTYAPFSSKDAKGEFIGFDIDLGNEMCKRMQVKCTWVASDFDALI<br>PSLKAKKIDAIISSLSITDKRQQEIAFSDKLYAADSRLIAAKGSPIQPTLESLKGKH<br>VGVLQGSTQEAYANDNWRTKGVDVVAYANQDLIYSDLTAGRLDAALQDEVAASEGFL<br>KQPAGKEYAFAGPSVKDKKYFGDGTGVGLRKDDTELKAAFDKALTELRQDGTYDKMA<br>KKYFDFNVYGDLEHHHHHH
	LAO.1	MALPQTVRIGTDTTYPPFSSKDAKGEFVGFDIDLGNEMCKRMQVKCTWVESDFDALI<br>PSLKAKKIDAIISSLSITDKRQQEIAFSDKLYAADARLIAPKGSPIQPTLESLKGKH<br>VGVLQGSTQEAYANDHWRTKGVDIVPYQNQDLIYSDLTAGRLDAALQDEVAASEGFL<br>KQPAGKDYAFAGPSVKDKKYFGDGTGVGLRKDDNELKAAFDKALAELRKDGTYDKMA<br>KKYFDFNVYGDLEHHHHHH
	LAO.2	MALPQTVRIGTDTTYPPFSSKDAQGQFVGFDIDLGNEMCKRMQVKCTWVESDFDALI<br>PSLKAKKIDAIISSLSITEKRQQEIAFSDKLYAADSRLIAPKGSPIQPTLESLKGKH<br>VGVLQGSTQEAYANDHWRSKGVDVVAYQNQDLIYSDLTAGRLDAALQDEVAASEGFL<br>KQPAGKDYAFAGPSVKDKKYFGDGTGVGLRKDDTELKAAFDKALAELRKDGTYDKMA<br>KKYFDFNVYGDLEHHHHHH
	LAO.3	MALPQTVRIGTDTTYPPFSSKDAKGEFVGFDIDLGNEMCKRMQVKCTWVESDFDALI<br>PSLKAKKIDAIISSLSITDKRQQEIAFSDKLYAADSRLIAPKGSPIQPTLESLKGKH<br>VGVLQGSTQEAYANDHWRTKGVDVVAYQNQDLIYSDLTAGRLDAALQDEVAASEGFL<br>KQPAGKDYAFAGPSVKDKKYFGDGTGVGLRKDDTELKAAFDKALAELRKDGTYDKMA<br>KKYFDFNVYGDLEHHHHHH
	LAO.4	MALPQTVRIGTDTTYAPFSSKDAKGEFVGFDIDLGNEMCKRMQVKCTWVESDFDALI<br>PSLKAKKIDAIISSLSITDKRQQEIAFSDKLYAADSRLIAPKGSPIQPTLESLKGKH<br>VGVLQGSTQEAYANDHWRTKGVDVVAYANQDLIYSDLTAGRLDAALQDEVAASEGFL<br>KQPAGKDYAFAGPSVKDKKYFGDGTGVGLRKDDTELKAAFDKALAELRKDGTYDKMA<br>KKYFDFNVYGDLEHHHHHH

Supporting Table 2. Amino acid sequences of PotF, TphC, MBP and LAO WT and designs tested in this study.

**Primer Name**	**DNA sequence**
TWIST amp for	CAATCCGCCCTCACTACAACCG
TphC_des2.0_term_rev	TTCGCTCACCCTCGAGTTACAACTTTAC
TphC_des2.1_term_rev	TTCGCTCACCCTCGAGTTATAACTTCAC
TphC_des2.2_term_rev	TTCGCTCACCCTCGAGTTAAAGCTTTAC
TphC_des2.3_term_rev	TTCGCTCACCCTCGAGTTATAACTTTAC
TphC_des2.4_term_rev	TTCGCTCACCCTCGAGTTAGAGCTTGAC

Supporting Table 3. Primers used in this study.

Protein target	Design ID	Apparent T_M in °C	ΔT_M in °C (- WT)	Apparent T_M in °C (ligand)	ΔT_M in °C (ligand - apo)
PotF	PotF.0	63.2 ± 0.40	0	70.3 ± 0.19	7.0 ± 0.59
	PotF.1	76.1 ± 0.32	12.9 ± 0.72	77.1 ± 0.26	1.0 ± 0.58
	PotF.2	72.7 ± 0.34	9.5 ± 0.74	75.5 ± 0.22	2.8 ± 0.56
	PotF.3	72.8 ± 0.34	9.5 ± 0.74	74.7 ± 0.17	1.9 ± 0.51
	PotF.4	74.6 ± 0.61	11.4 ± 1.01	75.5 ± 0.33	0.9 ± 0.94
TphC	TphC.0	60.6 ± 0.06	0	66.2 ± 0.04	5.7 ± 0.10
	TphC.1	67.9 ± 0.04	7.3 ± 0.10	69.7 ± 0.04	1.9 ± 0.08
	TphC.2	67.9 ± 0.05	7.3 ± 0.11	74.1 ± 0.03	6.2 ± 0.08
	TphC.3	66.4 ± 0.06	5.9 ± 0.12	72.5 ± 0.05	6.1 ± 0.11
	TphC.4	67.0 ± 0.05	6.4 ± 0.11	73.3 ± 0.07	6.3 ± 0.12
MBP	MBP.0	63.1 ± 0.09	0	68.0 ± 0.07	4.9 ± 0.16
	MBP.1	80.0 ± 0.13	16.9 ± 0.22	83.5 ± 0.13	3.5 ± 0.26
	MBP.2	79.1 ± 0.10	16.0 ± 0.19	80.2 ± 0.10	1.1 ± 0.20
	MBP.3	78.2 ± 0.12	15.1 ± 0.21	82.5 ± 0.10	4.3 ± 0.22
	MBP.4	77.5 ± 0.17	14.4 ± 0.26	81.0 ± 0.31	3.5 ± 0.48
LAO	LAO.0	47.5 ± 0.10	0	55.8 ± 0.07	8.4 ± 0.17
	LAO.1	51.4 ± 0.06	4.0 ± 0.16	55.9 ± 0.08	4.5 ± 0.14
	LAO.2	54.6 ± 0.08	7.2 ± 0.18	59.9 ± 0.06	5.3 ± 0.14
	LAO.3	53.7 ± 0.07	6.2 ± 0.17	58.1 ± 0.07	4.4 ± 0.14
	LAO.4	54.4 ± 0.22	6.9 ± 0.32	59.1 ± 0.16	4.7 ± 0.38

Supporting Table 4. Thermal melt data of all PBP variants.

Protein target	Design ID	Experimental repeat	Protein concentration (µM)	Ligand concentration (µM)
PotF	buffer-putrescine	1 - 3	-	600
	PotF.0	1 - 3	10.5	95
	PotF.1	1	15.2	120
		2 - 3	14.7	120
	PotF.2	1 - 3	14.8	120
	PotF.3	1 - 3	15.7	120
	PotF.4	1 - 3	16.2	120
TphC	buffer-terephthalate	1 - 3	-	278
	TphC.0	1 - 3	38.8	278
	TphC.1	1 - 2	59.0	278
		3	52.5	320
	TphC.2	1 - 3	51.1	278
	TphC.3	1 - 3	48.0	278
	TphC.4	1 - 3	31.2	200
	buffer-maltose	1 - 3	-	1000
	MBP.0	1 - 3	104.0	1000

MBP	MBP.1	1 - 2	70.4	500
		3	57.5	500
	MBP.2	1	98.9	900
		2	73.9	800
		3	97.9	1000
	MBP.3	1 - 3	99.9	1000
	MBP.4	1 - 3	96.3	1000
LAO	buffer-lysine	1 - 3	-	300
	LAO.0	1	24	200
		2 - 3	17	150
	LAO.1	1 - 3	71	500
	LAO.2	1	69	500
		2 - 3	38	500
	LAO.3	1	68	500
		2 - 3	60	500
	LAO.4	1 - 3	16	150

Supporting Table 5. Protein and ligand concentrations for ITC measurements

Protein target	Design ID	K_D (nM)	Δ*H* (kJ/mol)	-TΔ*S* (kJ/mol)	Δ*G* (kJ/mol)	N
PotF	PotF.0	42 ± 3	-88.9 ± 1.1	46.8 ± 1.2	-42.1 ± 0.2	0.92 ± 0.02
	PotF.1	479 ± 12	-76.2 ± 1.5	40.1 ± 1.6	-36.1 ± 0.1	0.97 ± 0.05
	PotF.2	42 ± 12	-87.3 ± 1.7	45.1 ± 2.5	-42.2 ± 0.8	0.92 ± 0.09
	PotF.3	101 ± 17	-82.7 ± 1.5	42.7 ± 1.1	-40.0 ± 0.4	0.95 ± 0.01
	PotF.4	103 ± 10	-88.8 ± 1.4	48.9 ± 0.4	-39.9 ± 0.2	0.88 ± 0.01
TphC	TphC.0	1180 ± 250	-43.1 ± 1.1	9.2 ± 1.5	-33.9 ± 0.5	0.95 ± 0.04
	TphC.1	5750 ± 709	-29.8 ± 3.9	-0.2 ± 4.1	-29.9 ± 0.3	0.88 ± 0.03
	TphC.2	627 ± 70	-36.1 ± 0.3	0.6 ± 0.6	-35.4 ± 0.3	0.92 ± 0.03
	TphC.3	716 ± 91	-36.7 ± 1.0	1.6 ± 1.3	-35.1 ± 0.3	0.92 ± 0.01
	TphC.4	282 ± 39	-45.6 ± 0.4	8.2 ± 0.7	-37.4 ± 0.4	0.97 ± 0.07
MBP	MBP.0	1240 ± 102	8.1 ± 0.0	-41.8 ± 0.2	-33.7 ± 0.2	0.96 ± 0.04
	MBP.1	1310 ± 293	-4.0 ± 0.3	-29.6 ± 0.8	-33.6 ± 0.5	1.15 ± 0.01
	MBP.2	< 10 μM	-	-	-	0.66 ± 0.03
	MBP.3	450 ± 44	3.8 ± 0.1	-40.1 ± 0.2	-36.2 ± 0.2	0.94 ± 0.03
	MBP.4	2960 ± 675	6.0 ± 0.3	-37.6 ± 0.4	-31.6 ± 0.6	1.02 ± 0.03
LAO	LAO.0	93 ± 35	-55.1 ± 0.9	14.9 ± 1.6	-40.2 ± 0.9	1.02 ± 0.01
	LAO.1	2496 ± 211	-20.2 ± 1.4	-11.8 ± 1.3	-32.0 ± 0.2	0.90 ± 0.00
	LAO.2	612 ± 56	-26.1 ± 0.2	-9.4 ± 0.1	-35.5 ± 0.2	0.91 ± 0.01
	LAO.3	824 ± 18	-25.4 ± 0.2	-9.3 ± 0.2	-34.7 ± 0.1	0.95 ± 0.03
	LAO.4	150 ± 21	-43.7 ± 0.5	4.7 ± 0.85	-39.0 ± 0.4	0.93 ± 0.04

Supporting Table 6. ITC data of all PBP variants. Averages and standard deviations were calculated based on three technical replicas.

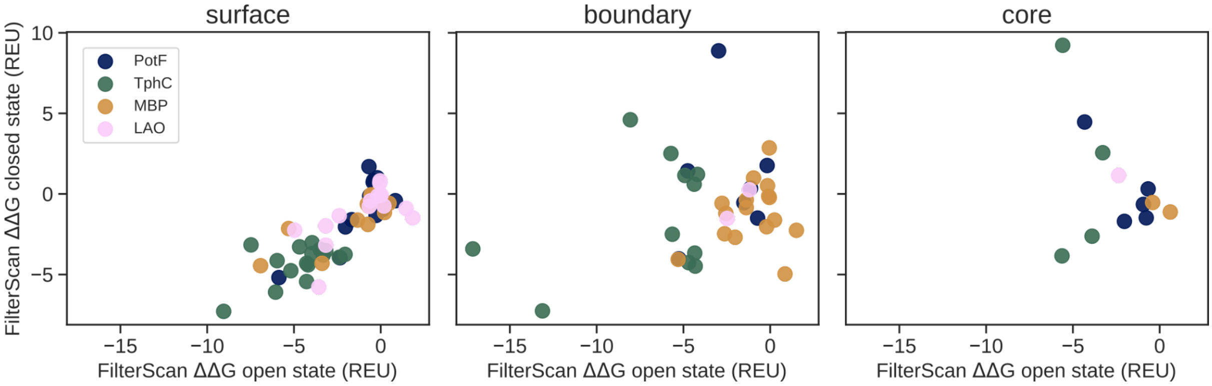

Supporting Figure 1. ΔΔ*G* values of PROSS mutations generated of PotF, TphC, MBP and LAO filtered by Rosetta LayerSelector categories: surface, boundary, and core. Open- and closed-state energies were plotted against each other.

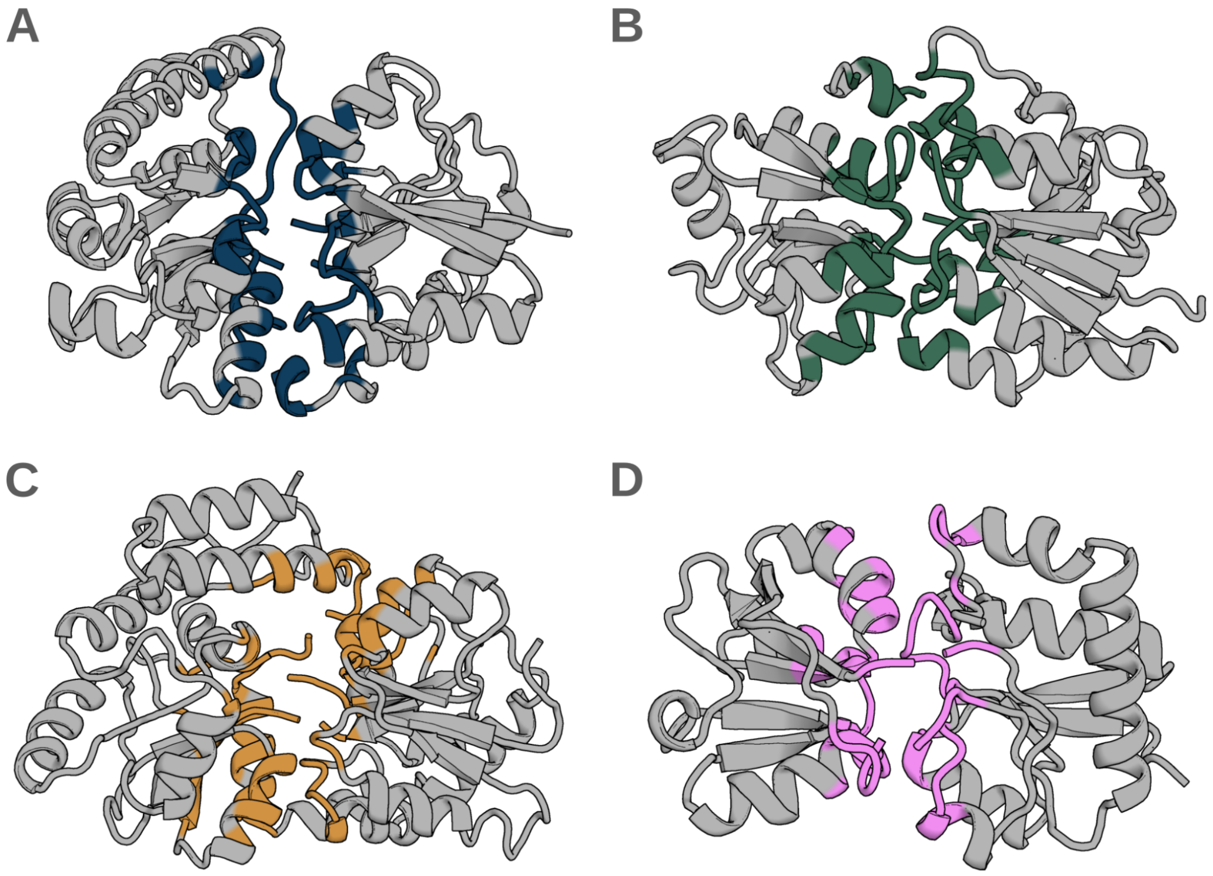

Supporting Figure 2. PBP interface residues that were fixed during PROSS design. (A-D) Closed-state PotF, TphC, MBP and LAO structures were split by predicted hinge residues and subdomain interface residues were highlighted in blue, green, orange and pink, respectively.

```
Positions 1-80
PotF.0    AEQKTLHIYNWSDYIAPDTVANFEKETGIKVVYDVFDSNEVLEGKLMAGSTGFDLVVPSASFLERQLTAGVFQPLDKSKL
PotF.1    AEQKTLHIYNWSDYIAPDTIANFEKETGIKVVYDVFDSNEVLEGKLMAGSTGFDLVVPSASFLERQLTAGVFQPLDKSKL
PotF.2    AEQKTLHIYNWSDYIAPDTVANFEKETGIKVVYDVFDSNEVLEGKLMAGSTGFDLVVPSASFLERQLTAGVFQPLDKSKL
PotF.3    AEQKTLHIYNWSDYIAPDTVANFEKETGIKVVYDVFDSNEVLEGKLMAGSTGFDLVVPSASFLERQLSAGVFQPLDKSKL
PotF.4    AEQKTLHIYNWSDYIAPDTVANFEKETGIKVVYDVFDSNEVLEGKLMAGSTGFDLVVPSASFLERQLTAGVFQPLDKSKL

Positions 81-160
PotF.0    PEWKNLDPELLKLVAKHDPDNKFAMPYMWATTGIGYNVDKVKAVLGENAPVDSWDLILKPENLEKLKSCGVSFLDAPEEV
PotF.1    PNWKNLDPELLKLVAQHDPDNKYAIPYMWATTGIGYNVDKVKAVLGEDAPVDSWDLILKPENLEKLKSCGVAFLDAPEEI
PotF.2    PNWKNLDPELLKLVAKHDPDNKYAMPYMWATTGIGYNVDKVKAVLGEDAPVDSWDLILKPENLEKLKSCGVAFLDAPEEI
PotF.3    PNWKNLDPELLKLVAKHDPDNKYAMPYMWATTGIGYNVDKVKAVLGEDAPVDSWDLILKPENLEKLKSCGVAFLDAPEEI
PotF.4    PNWKNLDPELLKLVAKHDPDNKYAMPYMWATTGIGYNVDKVKAVLGEDAPVDSWDLILKPENLEKLKSCGVAFLDAPEEI

Positions 161-240
PotF.0    FATVLNYLGKDPNSTKADDYTGPATDLLLKLRPNIRYFHSSQYINDLANGDICVAIGWAGDVWQASNRAKEAKNGVNVSF
PotF.1    FATVLNYLGKDPNSTNPDDYTGPATDLLLKLRPNIRYFHSSQYINDLANGDICVAIGWAGDVWQAANRAKEAKNGVNIAY
PotF.2    FATVLNYLGKDPNSTNADDYTGPATDLLLKLRPNIRYFHSSQYINDLANGDICVAIGWAGDVWQAANRAKEAKNGVNVAY
PotF.3    FATVLNYLGKDPNSTNADDYTGPATDLLLKLRPNIRYFHSSQYINDLANGDICVAIGWAGDVWQAANRAKEAKNGVNVAY
PotF.4    FATVLNYLGKDPNSTNADDYTGPATDLLLKLRPNIRYFHSSQYINDLANGDICVAIGWAGDVWQAANRAKEAKNGVNVAF

Positions 241-320
PotF.0    SIPKEGAMAFFDVFAMPADAKNKDEAYQFLNYLLRPDVVAHISDHVFYANANKAATPLVSAEVRENPGIYPPADVRAKLF
PotF.1    TIPKEGAMAFFDVFAMPADAKNKDEAYQFLNYLLRPDVMAHISDHVYYANANKAALPLVNPEVRDNPGIYPPPDVRAKLF
PotF.2    SIPKEGAMAFFDVFAMPADAKNKDEAYQFLNYLLRPDVVAHISNHVFYANANKAATSLVSAEVRDNPGIYPPPDVRAKLF
PotF.3    SIPKEGAMAFFDVFAMPADAKNKDEAYQFLNYLLRPDVVAHISDHVFYANANKAATPLVSAEVRDNPGIYPPPDVRAKLF
PotF.4    SIPKEGAMAFFDVFAMPADAKNKDEAYQFLNYLLRPDVVAHISDHVFYANANKAATALVSAEVRENPGIYPPADVRAKLF

Positions 321-352
PotF.0    TLKVQDPKIDRVRTRAWTKVKSGKLEHHHHHH
PotF.1    TLKVQDPKIDRVRTRAWTKVKSGKLEHHHHHH
PotF.2    TLKVQDPKIDRVRTRAWTKVKSGKLEHHHHHH
PotF.3    TLKVQDPKIDRVRTRAWTKVKSGKLEHHHHHH
PotF.4    TLKVQDPKIDRVRTRAWTKVKSGKLEHHHHHH
```

Supporting Figure 3. Amino acid sequence alignment of PotF.0-PotF.4 with mutations highlighted. Additionally, hinge and interface residues are shown as grey boxes.

Positions 1-80

```
TphC.0    GSSHHHHHHGSGENLYFQSNQPLKIVVPFSAGGTADVLPRLVAEKIRADYAGGVIIENKPGAGGNIGADLVFRAPPDGMT
TphC.1    GSSHHHHHHGSGENLYFQSNQPLKIVVPFSPGGTADVLPRLVAEKIRADYGGGVIIENKPGAGGNIGADLVFRAPPDGYT
TphC.2    GSSHHHHHHGSGENLYFQSNQPLKIVVPFSPGGTADVLPRLVAEKIRKDYAGGVIIENKPGAGGNIGADLVFRAPPDGMT
TphC.3    GSSHHHHHHGSGENLYFQSNQPLKIVVPFSPGGTADVLPRLVAEKIRADYAGGVIIENKPGAGGNIGADLVFRAPPDGMT
TphC.4    GSSHHHHHHGSGENLYFQSNQPLKIVVPFSAGGTADVLPRLVAEKIRADYAGGVIIENKPGAGGNIGADLVFRAPPDGYT
```

Positions 81-160

```
TphC.0    VLASPPGPIAINHNLYQKLSFDPTRWVPVTILATVPNVLVINPKLPVKSLGEFIAYAKANPKKVTVATQGDGSTSHLTAA
TphC.1    VLISPPGPIAINHNLYKKLSFDPTRWVPVTILATVPNVLVINPKLPVKSVKEFIAYAKANPGKLTVATQGNGSTSHLTAE
TphC.2    VLASPPGPIAINHNLYKKLSFDPTRWVPVTILATVPNVLVINPKLPVKSVKEFIAYAKANPGKVTVATQGNGSTSHLTAA
TphC.3    VLASPPGPIAINHNLYKKLSFDPTRWVPVTILATVPNVLVINPKLPVKSVKEFIAYAKANPGKVTVATQGNGSTSHLTAA
TphC.4    VLASPPGPIAINHNLYQKLSFDPTRWVPVTILATVPNVLVINPKLPVKSLKEFIAYAKANPGKVTVATQGDGSTSHLTAA
```

Positions 161-240

```
TphC.0    MFMQLTGTELTVIPYKGTAPALIDLIGGNVDVFFDNISSSATYHQAGKVRILAVADEQRSQILPQVPTFAEQQWPAMQAV
TphC.1    LFMQMTGTDMTHIPYKGTAPALIDLIGGNVDVFFDNISSSATYHQAGKVRILAVADEQRSPILPDVPTFAEQGFPAFQVV
TphC.2    MFMQLTGTDMTHIPYKGTAPALIDLIGGNVDVFFDNISSSAQYHQAGKVRILAVADEQRSPILPDVPTFAEQGFPAMQAV
TphC.3    MFMQLTGTDMTHIPYKGTAPALIDLIGGNVDVFFDNISSSATYHQAGKVRILAVADEQRSPILPDVPTFAEQGFPAMQAV
TphC.4    MFMQLTGTELTVIPYKGTAPALIDLIGGNVDVFFDNISSSATYHQAGKVRILAVADEQRSQILPQVPTFAEQGFPAMQAV
```

Positions 241-313

```
TphC.0    TFFSVVAPPGTSAEIAQKLQKQMALALSSNDIRKHFQEQGAVPCGWDPSKTAQFIRQETEKWKKVLKAANVKL
TphC.1    TFFSVVAPPGTSAEIVQKLQKQIAKALSSPDIRKHFQEQGAVPVGWDPSQTAQFIRQETEKWKKVIKAANVKL
TphC.2    TFFSVVAPPGTSAEIAQKLQKQMAKALSSPDIRKHFQEQGAVPVGWDPEQTAQFIRQETEKWKKVLKAANVKL
TphC.3    TFFSVVAPPGTSAEIAQKLQKQMAKALSSPDIRKHFQEQGAVPVGWDPSQTAQFIRQETEKWKKVLKAANVKL
TphC.4    TFFSVVAPPGTSAEIAQKLQKQMAKALSSPDIRKHFQEQGAVPCGWDPEQTAQFIRQETEKWKKVLKAANVKL
```

Supporting Figure 4. Amino acid sequence alignment of TphC.0-TphC.4 with mutations highlighted. Additionally, hinge and interface residues are shown as grey boxes.

Positions 1-80

```
MBP.0    KIEEGKLVIWINGDKGYNGLAEVGKKFEKDTGIKVTVEHPDKLEEKFPQVAATGDGPDIIFWAHDRFGGYAQSGLLAEIT
MBP.1    KIEEGKLVIWINGDKGYNGLAEVGKKFEKDTGIKVTVEHPDKLEEKFAQVAATGDGPDIIFWAHDRFGGWAQSGLLAEIH
MBP.2    KIEEGKLVIWINGDKGYNGLAEVGKKFEKDTGIKVTVEHPDKLEEKFTQVAATGDGPDIIFWAHDRFGGWAQSGLLAEIH
MBP.3    KIEEGKLVIWINGDKGYNGLAEVGKKFEKDTGIKVTVEHPDKLEEKFAQVAATGDGPDIIFWAHDRFGGWAQSGLLAEIH
MBP.4    KIEEGKLVIWINGDKGYNGLAEVGKKFEKDTGIKVTVEHPDKLEEKFAQVAATGDGPDIIFWAHDRFGGYAQSGLLAEIT
```

Positions 81-160

```
MBP.0    PDKAFQDKLYPFTWDAVRYNGKLIAYPIAVEALSLIYNKDLLPNPPKTWEEIPALDKELKAKGKSALMFNLQEPYFTWPL
MBP.1    PDKEFQDKLFPFTWDAVRYNGKLIAYPIAVEALSLIYNKDLLPNPPKTWEELPELDKKLRAKGKSAIMFNLQEPYFTWPL
MBP.2    PDKAFQDKFYPFTWDAVRYNGKLIAYPVAVEALSLIYNKDLLPNPPKTWEELPELDKELRKKGKSALMFNLQEPYFTWPL
MBP.3    PDKAFQDKLYPFTWDAVRYNGKLIAYPIAVEALSLIYNKDLLPNPPKTWEELPELDKELRAKGKSALMFNLQEPYFTWPL
MBP.4    PDKAFQDKFYPFTWDAVRYNGKLIAYPIAVEALSLIYNKDLLPNPPKTWEELPALDKELRAKGKSALMFNLQEPYFTWPL
```

Positions 161-240

```
MBP.0    IAADGGYAFKYENGKYDIKDVGVDNAGAKAGLTFLVDLIKNKHMNADTDYSIAEAAFNKGETAMTINGPWAWSNIDTSKV
MBP.1    IAADGGYAFKYENGKYDIKDVGVNNEGAKAGLQFLVDLIKNKHMNADIDYSIAEAAFNKGETAMTINGPWAWSNIDKAKI
MBP.2    IAADGGYAFKYENGKYDIKDVGVNNEGAKAGLQFLVDLIKNKHMNADIDYSIAEAAFNKGETAMTINGPWAWSNIDKAKI
MBP.3    IAADGGYAFKYENGKYDIKDVGVNNEGAKAGLQFLVDLIKNKHMNADIDYSIAEAAFNKGETAMTINGPWAWSNIDKAKI
MBP.4    IAADGGYAFKYENGKYDIKDVGVNNEGAKAGLQFLVDLIKNKHMNADTDYSIAEAAFNKGETAMTINGPWAWSNIDKAKI
```

Positions 241-320

```
MBP.0    NYGVTVLPTFKGQPSKPFVGVLSAGINAASPNKELAKEFLENYLLTDEGLEAVNKDKPLGAVALKSYEEELAKDPRIAAT
MBP.1    NYGVTVLPTFKGKPSKPFVGVLSAGINAASPNKELAKEFLENYLLTDEGLDLVNKDKPLGAVALKSYQEELAKDPRIAAT
MBP.2    NYGVTVLPTFKGKPSKPFVGVLTAGINAASPNKELAKEFLENYLLTDEGLDMVNKDKPLGAVALKSYQEELAKDPRIAAT
MBP.3    NYGVTVLPTFKGKPSKPFVGVLSAGINAASPNKELAKEFLENYLLTDEGLDAVNKDKPLGAVALKSYQEELAKDPRIAAT
MBP.4    NYGVTVLPTFKGKPSKPFVGVLSAGINAASPNKELAKEFLENYLLTDEGLDAVNKDKPLGAVALKSYQEELAKDPRIAAT
```

Positions 321-378

```
MBP.0    MENAQKGEIMPNIPQMSAFWYAVRTAVINAASGRQTVDEALKDAQTRITKLEHHHHHH
MBP.1    MENAQNGEIMPNIPQMSAFWYAMRTAVINALSGRQSVDEALKDAQTRITKLEHHHHHH
MBP.2    MENAQNGEIMPNIPQMSAFWYAMRTAVINALSGRQTVDEALKDAQKRITKLEHHHHHH
MBP.3    MENAQNGEIMPNIPQMSAFWYAMRTAVINALSGRQTVDEALKDAQTRITKLEHHHHHH
MBP.4    MENAQKGEIMPNIPQMSAFWYAMRTAVINALSGRQTVDEALKDAQTRITKLEHHHHHH
```

Supporting Figure 5. Amino acid sequence alignment of MBP.0-MBP.4 with mutations highlighted. Additionally, hinge and interface residues are shown as grey boxes.

```
Positions 1-80
LAO.0    ALPQTVRIGTDTTYAPFSSKDAKGEFIGFDIDLGNEMCKRMQVKCTWVASDFDALIPSLKAKKIDAIISSLSITDKRQQE
LAO.1    ALPQTVRIGTDTTYPPFSSKDAKGEFVGFDIDLGNEMCKRMQVKCTWVESDFDALIPSLKAKKIDAIISSLSITDKRQQE
LAO.2    ALPQTVRIGTDTTYPPFSSKDAQGQFVGFDIDLGNEMCKRMQVKCTWVESDFDALIPSLKAKKIDAIISSLSITEKRQQE
LAO.3    ALPQTVRIGTDTTYPPFSSKDAKGEFVGFDIDLGNEMCKRMQVKCTWVESDFDALIPSLKAKKIDAIISSLSITDKRQQE
LAO.4    ALPQTVRIGTDTTYAPFSSKDAKGEFVGFDIDLGNEMCKRMQVKCTWVESDFDALIPSLKAKKIDAIISSLSITDKRQQE

Positions 81-160
LAO.0    IAFSDKLYAADSRLIAAKGSPIQPTLESLKGKHVGVLQGSTQEAYANDNWRTKGVDVVAYANQDLIYSDLTAGRLDAALQ
LAO.1    IAFSDKLYAADARLIAPKGSPIQPTLESLKGKHVGVLQGSTQEAYANDHWRTKGVDIVPYQNQDLIYSDLTAGRLDAALQ
LAO.2    IAFSDKLYAADSRLIAPKGSPIQPTLESLKGKHVGVLQGSTQEAYANDHWRSKGVDVVAYQNQDLIYSDLTAGRLDAALQ
LAO.3    IAFSDKLYAADSRLIAPKGSPIQPTLESLKGKHVGVLQGSTQEAYANDHWRTKGVDVVAYQNQDLIYSDLTAGRLDAALQ
LAO.4    IAFSDKLYAADSRLIAPKGSPIQPTLESLKGKHVGVLQGSTQEAYANDHWRTKGVDVVAYANQDLIYSDLTAGRLDAALQ

Positions 161-240
LAO.0    DEVAASEGFLKQPAGKEYAFAGPSVKDKKYFGDGTGVGLRKDDTELKAAFDKALTELRQDGTYDKMAKKYFDFNVYGDLE
LAO.1    DEVAASEGFLKQPAGKDYAFAGPSVKDKKYFGDGTGVGLRKDDNELKAAFDKALAELRKDGTYDKMAKKYFDFNVYGDLE
LAO.2    DEVAASEGFLKQPAGKDYAFAGPSVKDKKYFGDGTGVGLRKDDTELKAAFDKALAELRKDGTYDKMAKKYFDFNVYGDLE
LAO.3    DEVAASEGFLKQPAGKDYAFAGPSVKDKKYFGDGTGVGLRKDDTELKAAFDKALAELRKDGTYDKMAKKYFDFNVYGDLE
LAO.4    DEVAASEGFLKQPAGKDYAFAGPSVKDKKYFGDGTGVGLRKDDTELKAAFDKALAELRKDGTYDKMAKKYFDFNVYGDLE

Positions 241-246
LAO.0    HHHHHH
LAO.1    HHHHHH
LAO.2    HHHHHH
LAO.3    HHHHHH
LAO.4    HHHHHH
```

Supporting Figure 6. Amino acid sequence alignment of LAO.0-LAO.4 with mutations highlighted. Additionally, hinge and interface residues are shown as grey boxes.

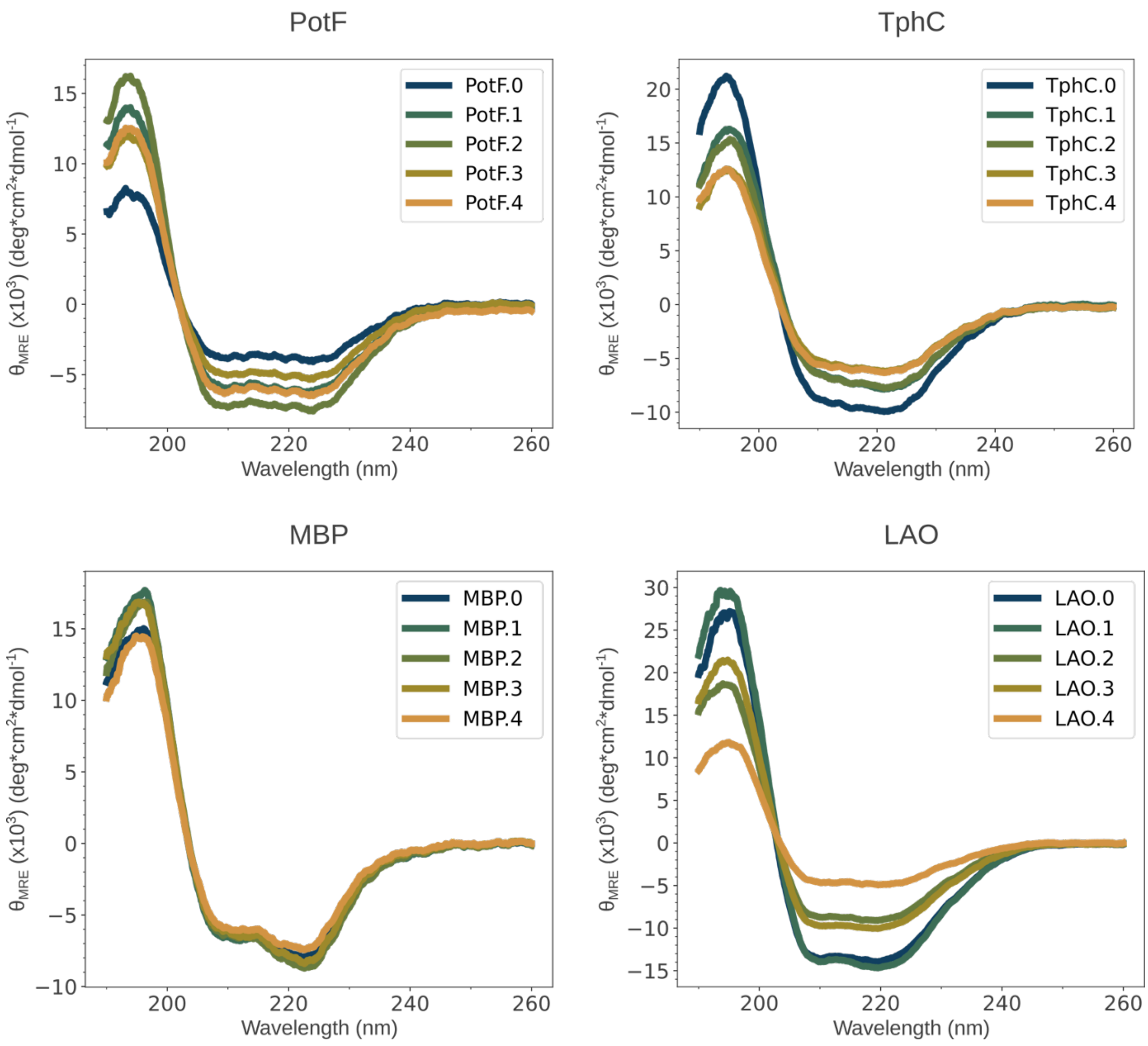

Supporting Figure 7. CD spectra of all PBP variants without ligands present plotted as mean residue ellipticity (θ_{MRE}) against the wavelength.

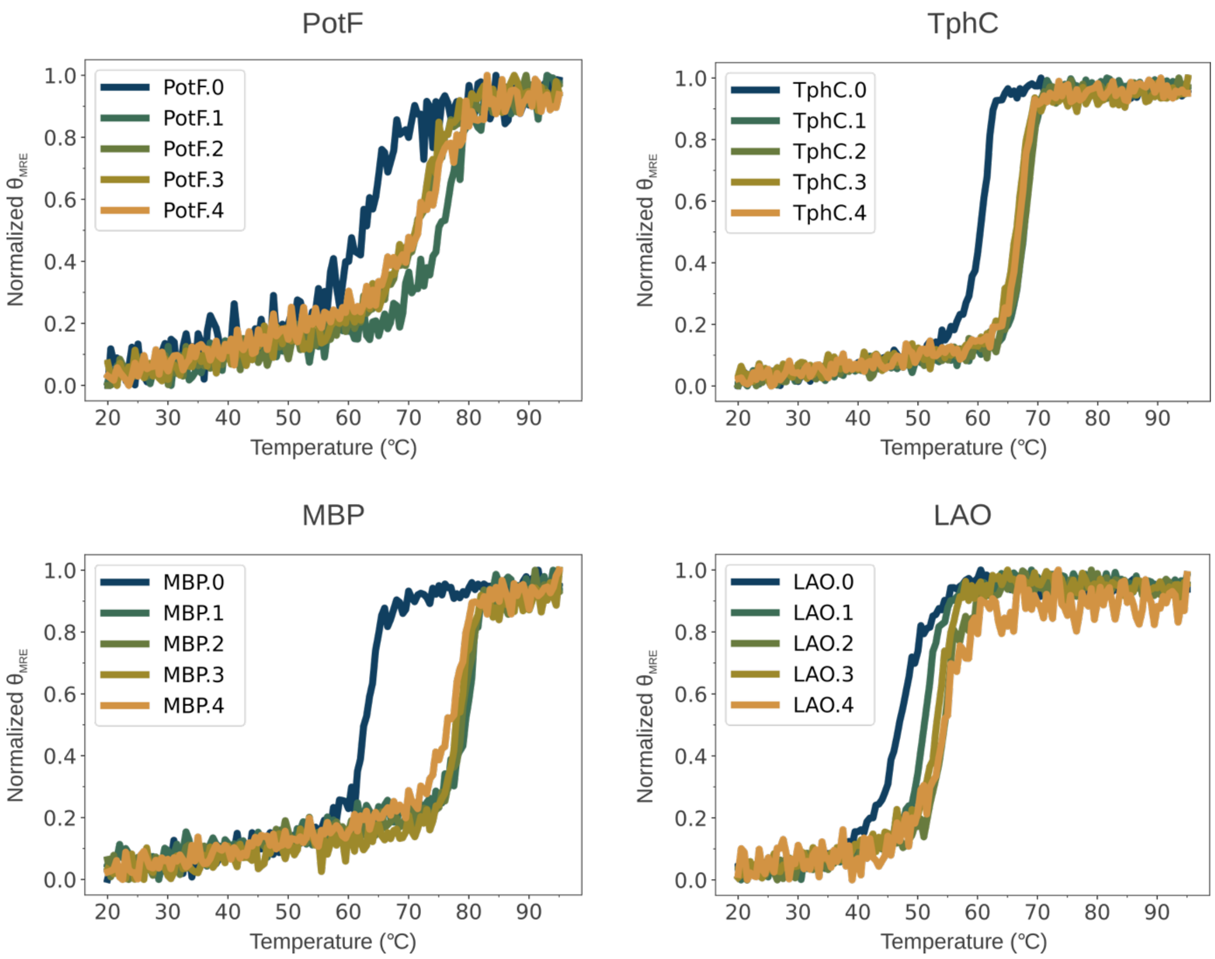

Supporting Figure 8. CD thermal melts of all PBP variants without ligands present plotted as normalized mean residue ellipticity (θ_{MRE}) against a temperature gradient.

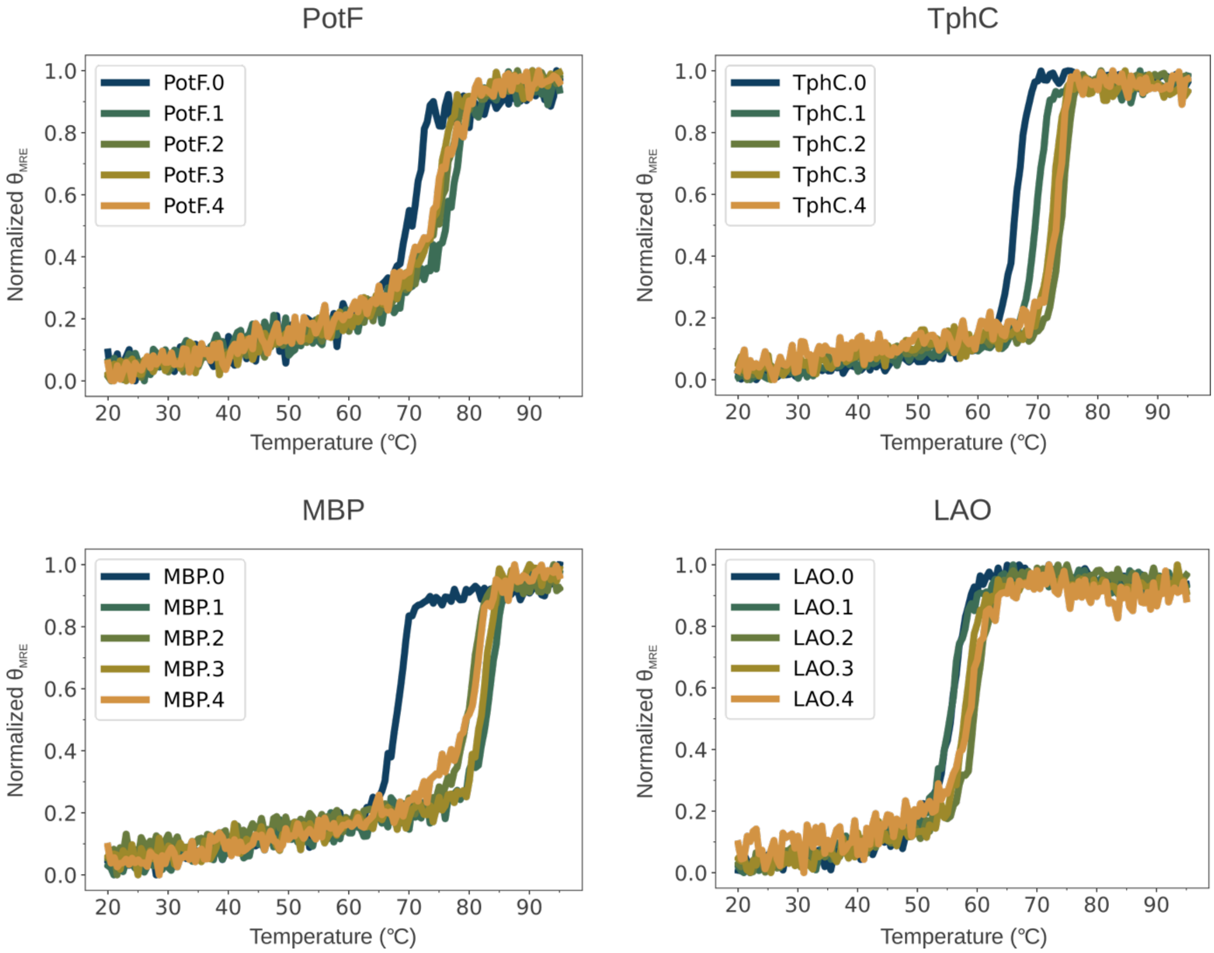

Supporting Figure 9. CD thermal melts of all PBP variants with ligands present plotted as normalized mean residue ellipticity (θ_{MRE}) against a temperature gradient.

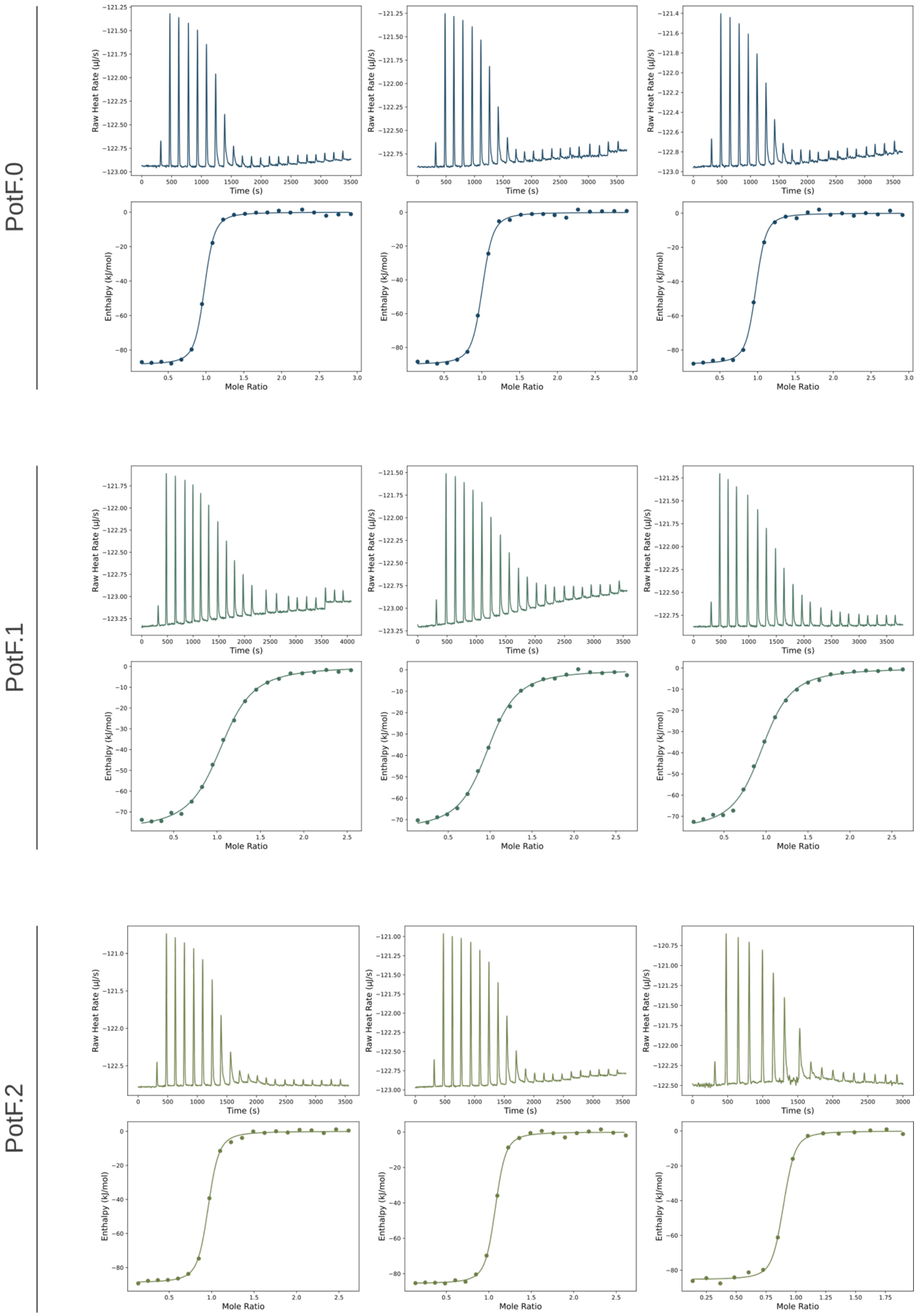
PotF.0
PotF.1
PotF.2
Raw Heat Rate (μJ/s)
Time (s)
Enthalpy (kJ/mol)
Mole Ratio

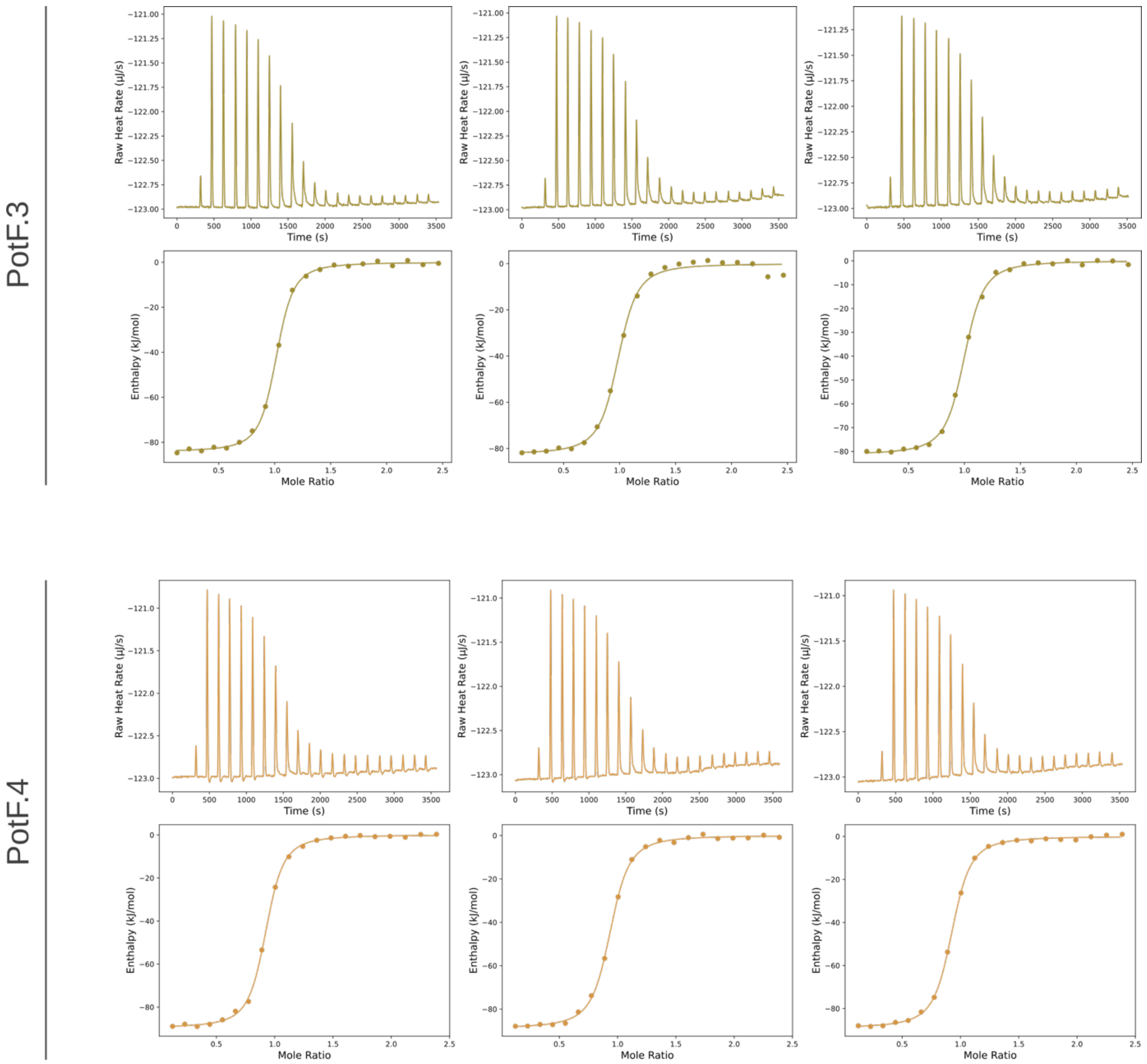

Supporting Figure 10. Thermograms and binding isotherms of PotF.0-4 and putrescine in three technical replicates. Buffer-putrescine control titrations were performed to obtain baseline values that were subtracted from the integrated heats of PotF.0-4.

TphC.0

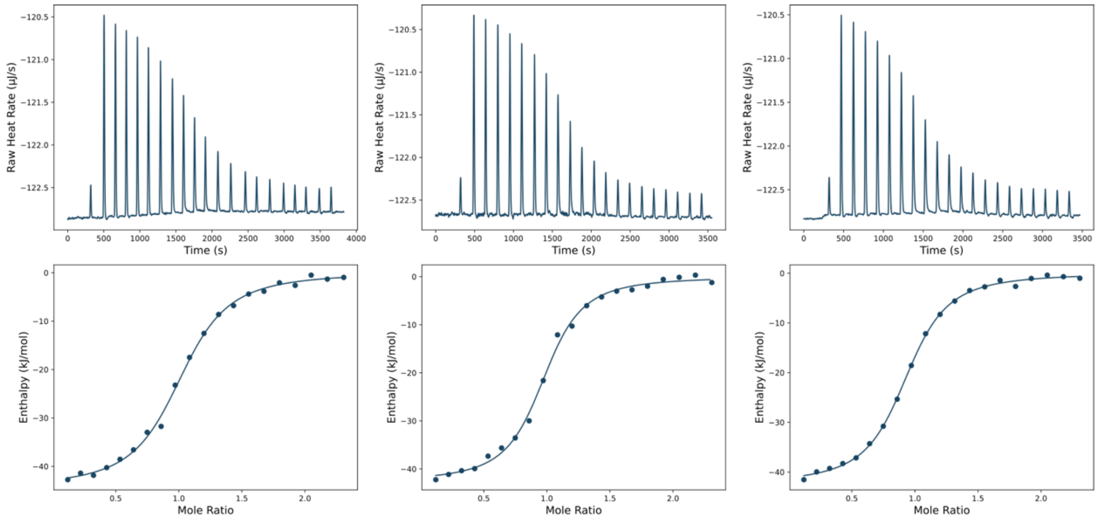

TphC.1

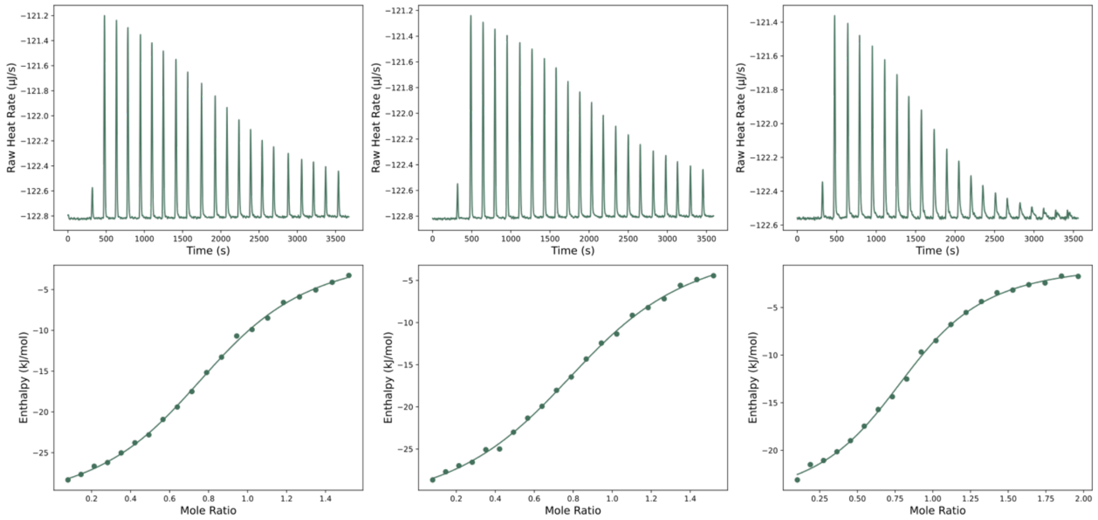

TphC.2

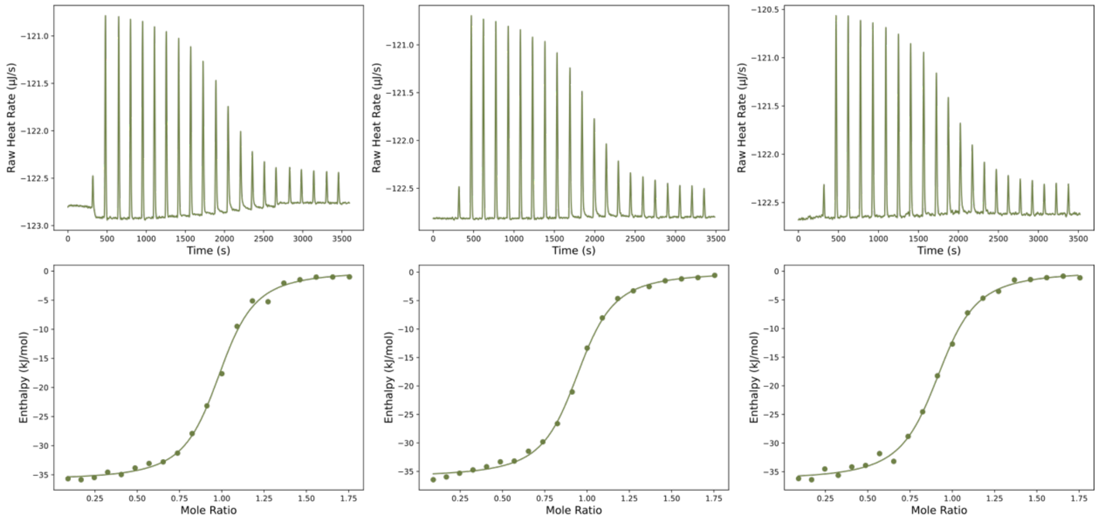

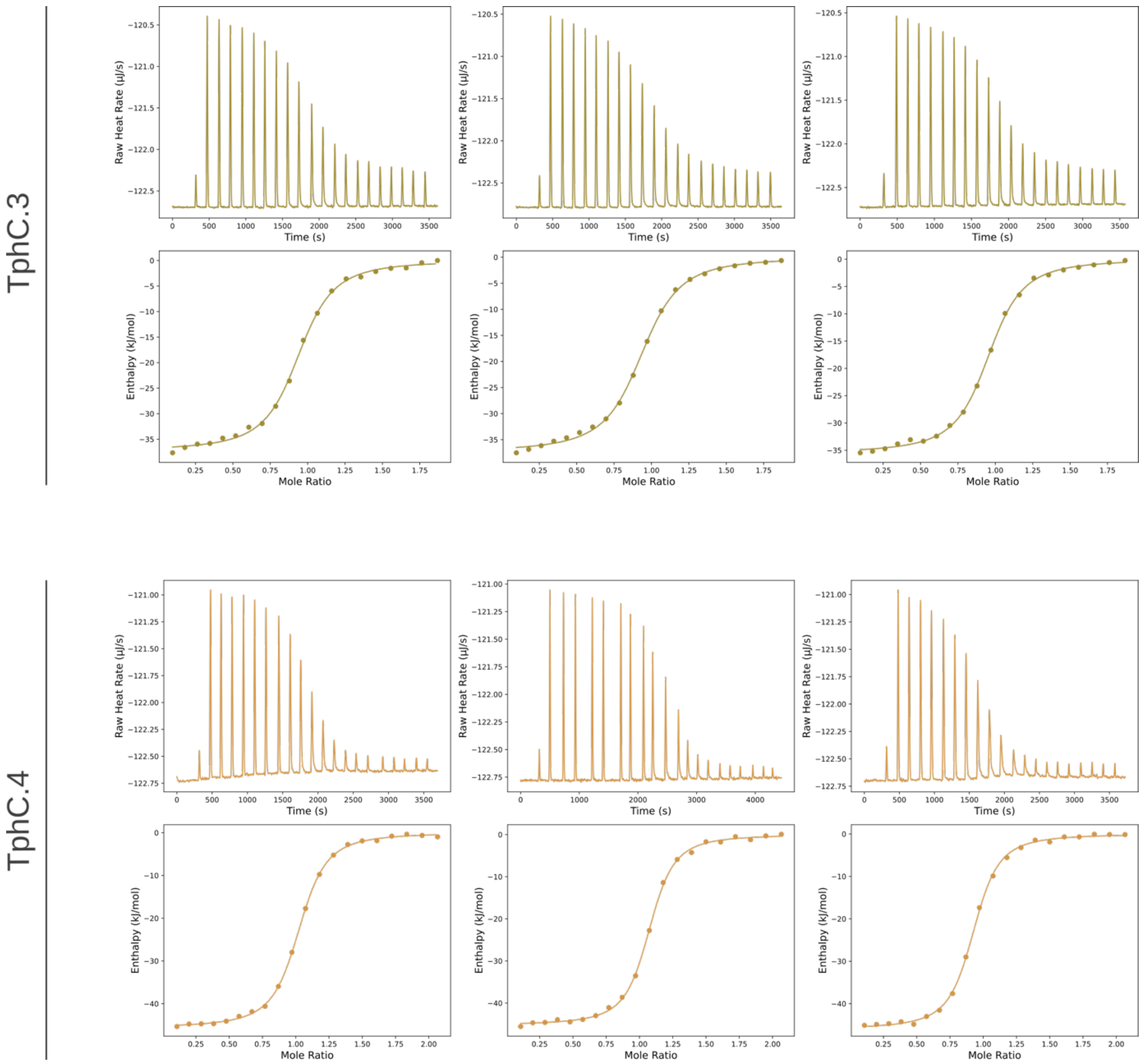

Supporting Figure 11. Thermograms and binding isotherms of TphC.0-4 and terephthalate in three technical replicates. Buffer-terephthalate control titrations were performed to obtain baseline values that were subtracted from the integrated heats of TphC.0-4.

MBP.0

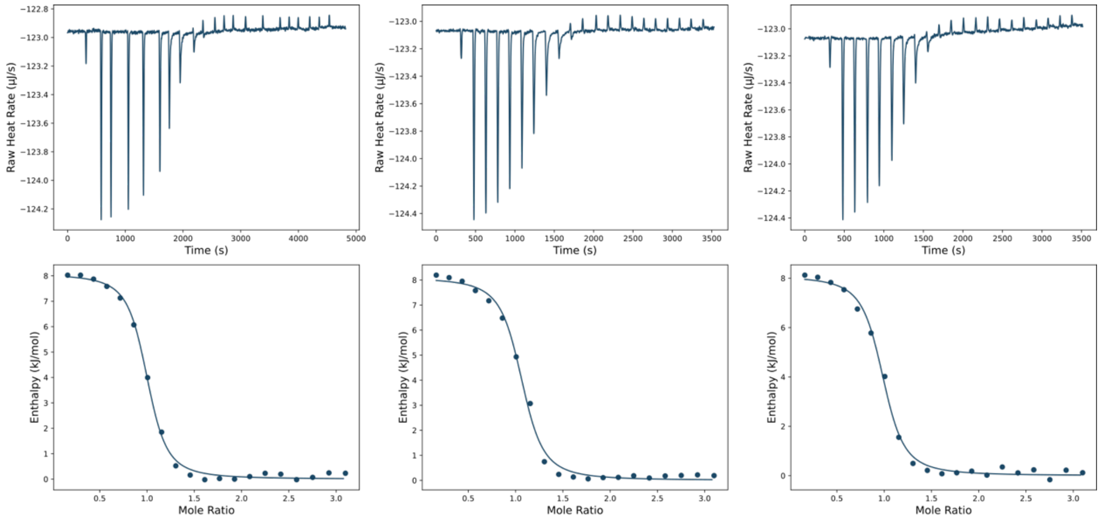

MBP.1

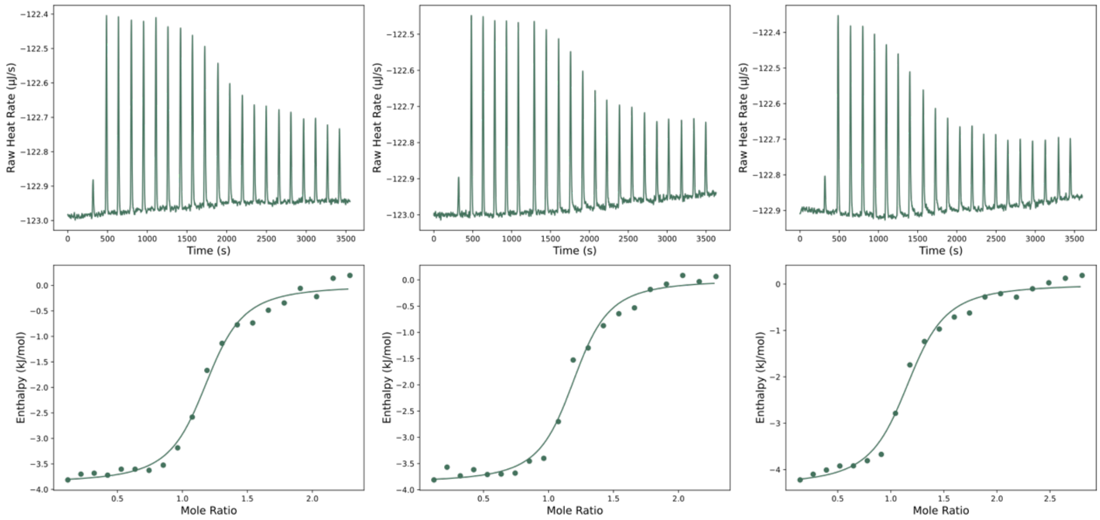

MBP.2

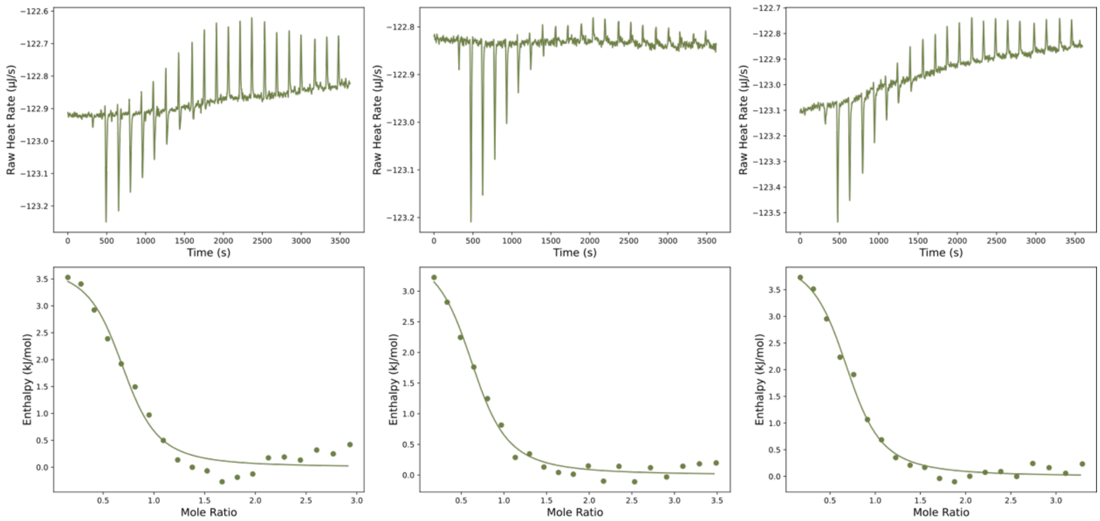

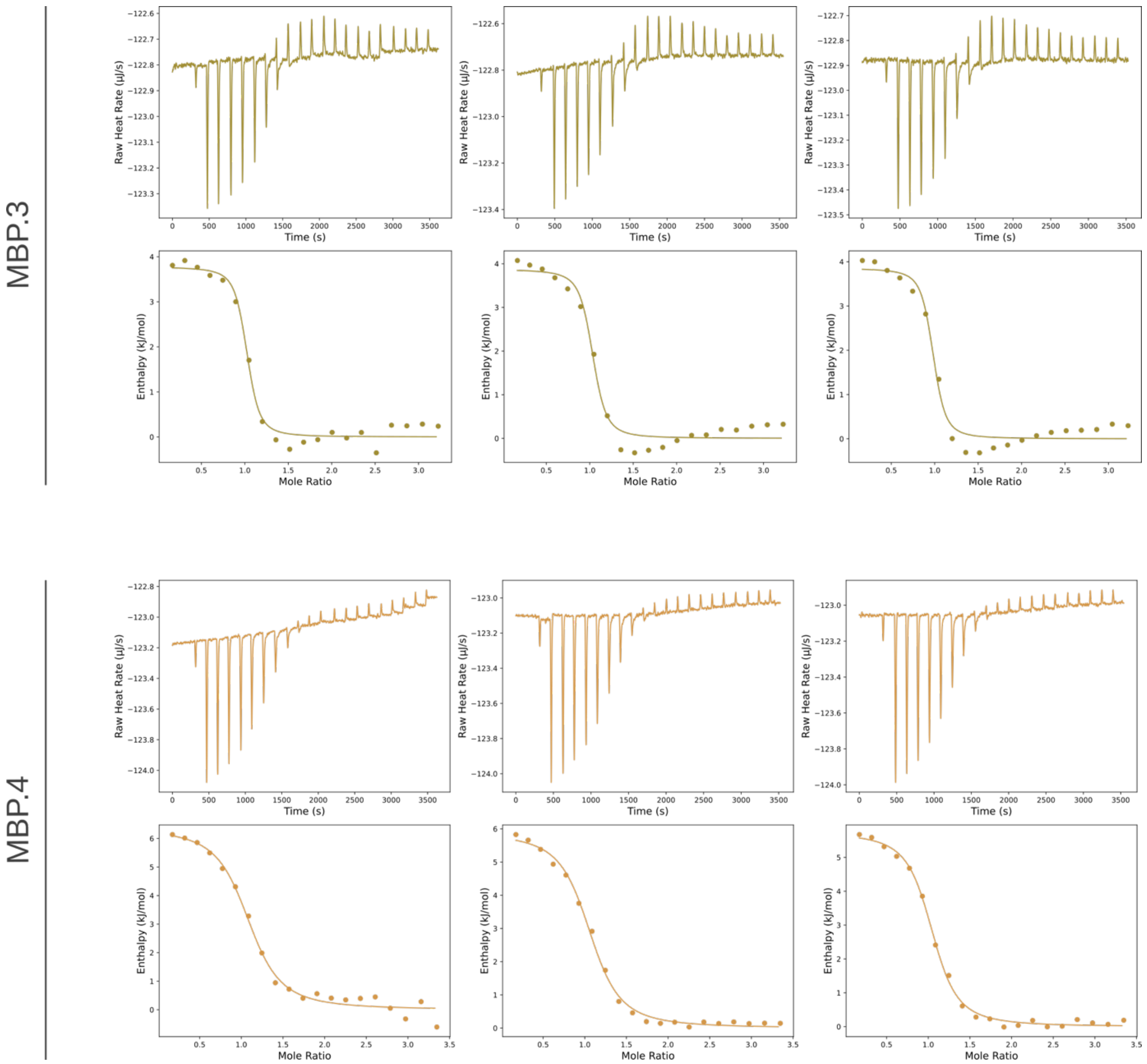

Supporting Figure 12. Thermograms and binding isotherms of MBP.0-4 and maltose in three technical replicates. Buffer-maltose control titrations were performed to obtain baseline values that were subtracted from the integrated heats of MBP.0-4.

LAO.0

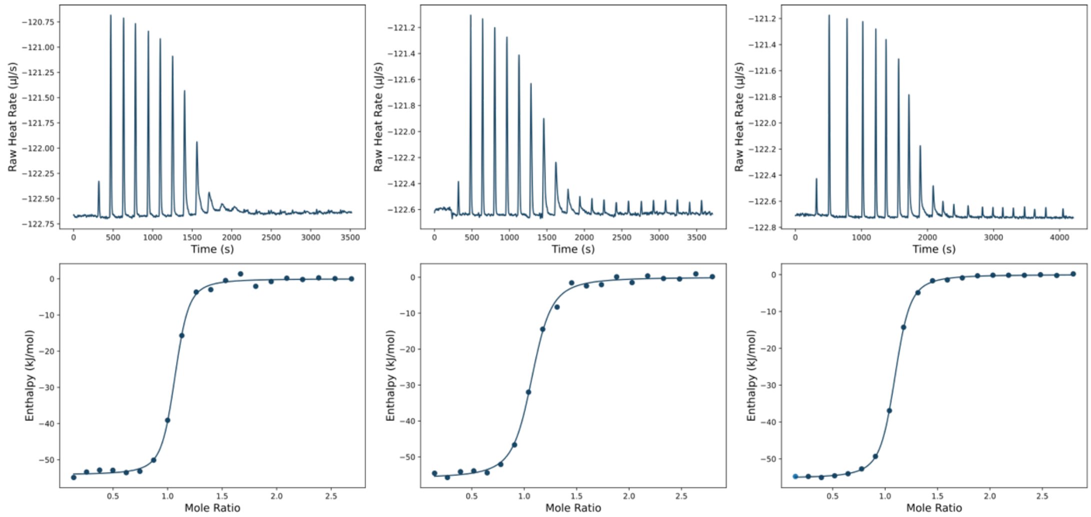

LAO.1

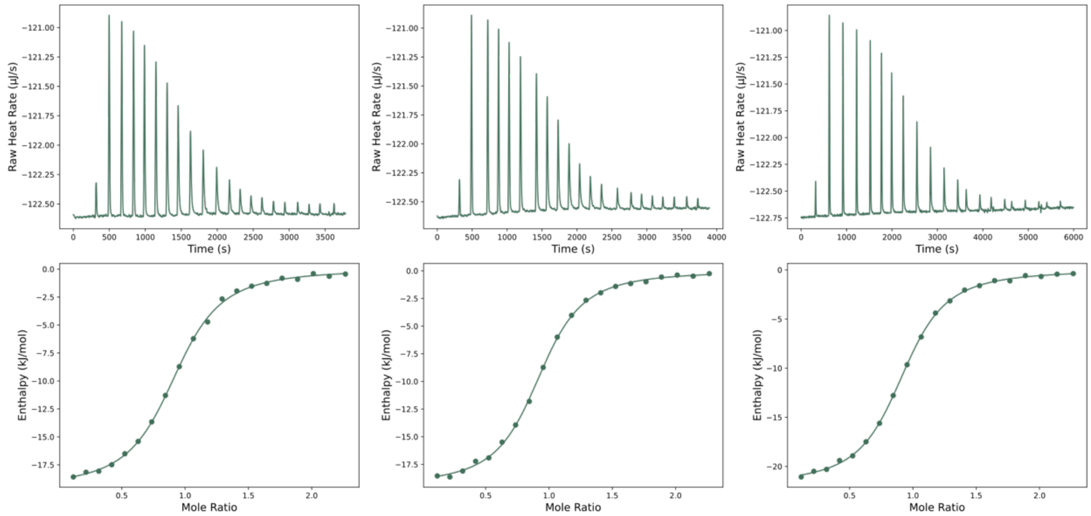

LAO.2

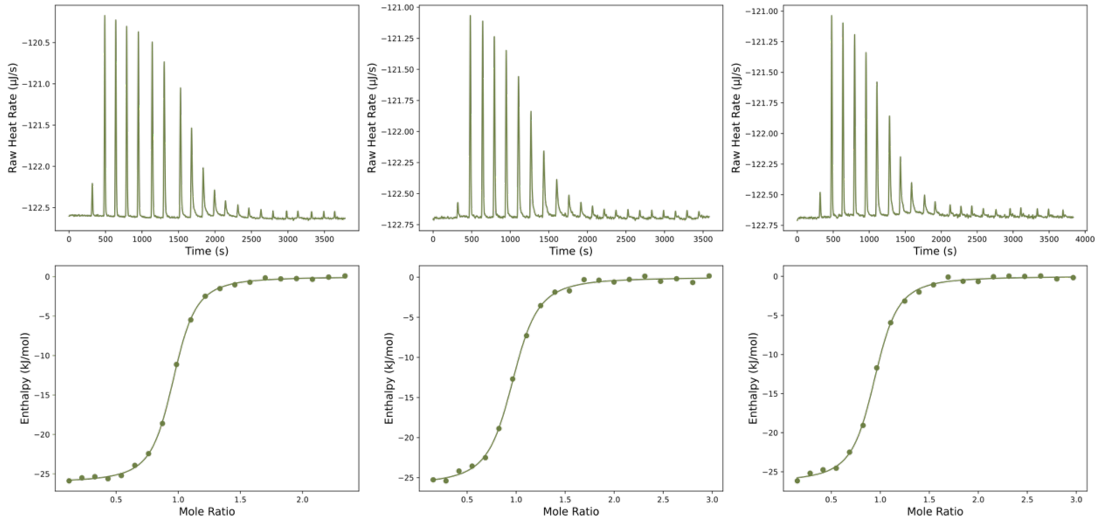

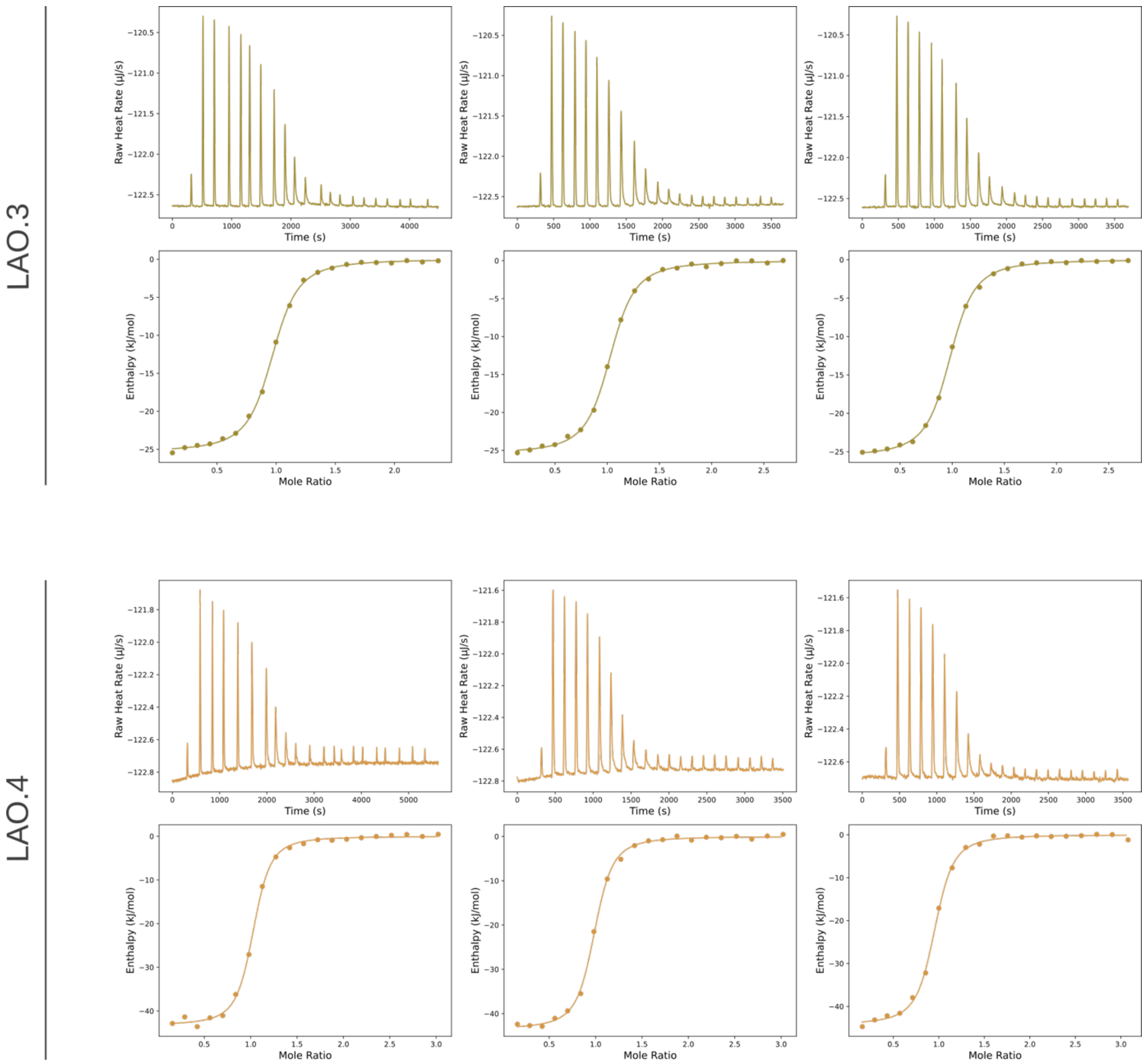

Supporting Figure 13. Thermograms and binding isotherms of LAO.0-4 and L-lysine in three technical replicates. Buffer-lysine control titrations were performed to obtain baseline values that were subtracted from the integrated heats of LAO.0-4.

References

1. Kröger, P., Shanmugaratnam, S., Ferruz, N., Schweimer, K. & Höcker, B. A comprehensive binding study illustrates ligand recognition in the periplasmic binding protein PotF. *Structure* **29**, 433-443.e4 (2021).
2. Gautom, T. *et al.* Structural basis of terephthalate recognition by solute binding protein TphC. *Nat. Commun.* **12**, 6244 (2021).
3. Sharff, A. J., Rodseth, L. E., Spurlino, J. C. & Quiocho, F. A. Crystallographic evidence of a large ligand-induced hinge-twist motion between the two domains of the maltodextrin binding protein involved in active transport and chemotaxis. *Biochemistry* **31**, 10657–10663 (1992).
4. Quiocho, F. A., Spurlino, J. C. & Rodseth, L. E. Extensive features of tight oligosaccharide binding revealed in high-resolution structures of the maltodextrin transport/chemosensory receptor. *Structure* **5**, 997–1015 (1997).
5. Oh, B. H. *et al.* Three-dimensional structures of the periplasmic lysine/arginine/ornithine-binding protein with and without a ligand. *J. Biol. Chem.* **268**, 11348–11355 (1993).
6. Oh, B. H., Ames, G. F. & Kim, S. H. Structural basis for multiple ligand specificity of the periplasmic lysine-, arginine-, ornithine-binding protein. *J. Biol. Chem.* **269**, 26323–26330 (1994).
7. Berman, H. M. *et al.* The Protein Data Bank. *Nucleic Acids Res.* **28**, 235–242 (2000).
